# Hamlet’s Apostrophe to Man

**Published:** 1893-04

**Authors:** 


### HOMŒOPATHIC PHYSICIAN,

A MONTHLY JOURNAL OF

HOMŒOPATHIC MATERIA MEDICA AND CLINICAL MEDICINE.

"If our school ever gives up the strict inductive method of Hahnemann, we are lost, and deserve only to be mentioned as a caricature in the history of medicine."—CONSTANTINE HERING.

Vol. XIII. APRIL, 1893. No. 4.

### EDITORIAL.

HAMLET'S APOSTROPHE TO MAN.—"What a piece of work is man! How noble in reason! how infinite in faculty! in form and moving how express and admirable! in action how like an angel! in apprehension how like a god! the beauty of the world! the paragon of animals!"

This brilliant apostrophe of the immortal Shakespeare to man, so well known to every lover of the play of Hamlet, must strike a responsive thrill in every human heart. For in our adoration of ourselves and our consequent admiration and pride in humanity in general, we are all optimists. In the contemplation of it our self-esteem is satisfied, our vanity gratified. Meaner minds show the vividness of this sentiment within them by their offensive self-assertion, and their contempt for the lower animals; not hesitating to visit upon the latter cruelties and outrages the most atrocious without the slightest misgiving of the unrighteousness of the act or the possibility of retribution. Nobler minds, under the influence of the same sentiment, call up in imagination the great concourse of brilliant men who have figured in the world's history: warriors and statesmen, poets and musicians, sculptors and painters, inventors and discoverers.

But every medal has its reverse; every sunlit landscape its shadows. If we remember with enthusiasm the achievements of great men, especially those who have most benefited the world, we must also remember the fierce struggles which nearly all of them have experienced in order to get their ideas understood, to say nothing of having them accepted.

All of us are familiar with the stories of many of these men, and we recall with the crimson blush of shame and anger the persecutions of the founder of our system of medicine.

The human mind is so far the creature of habit, it is so deeply steeped in indolence, it is so incapable of investigation, it is so troubled with timidity that it has an instinctive aversion to any new idea implying change and the abandonment of established routine. This constitutes the *littleness* of the human mind, and renders it more subservient to those natural laws which govern lesser organizations. The Darwinian law of gradual evolution which pervades the animal and vegetable kingdoms generally governs it almost to the exclusion of its Divine intelligence and judgment, so that whatever principle of progress or elevation is presented to it must have traveled step by step through the most painful and vexatious stages of delay before it will accept it. When, on the other hand, an idea, however grand and true and noble, springs ready-formed from the brain of some brilliant man, and, ignoring the slow steps of evolution, stands forth claiming recognition, its temerity in disobeying this law of evolution is promptly punished by a refusal to give it consideration. The human intellect objects to listen or it interrupts with frivolous commonplaces; or it disputes with exasperating sophistry, or it persecutes savagely, and so it manages to rear an intellectual Chinese wall of obstruction by which it shuts out all knowledge of new ideas, new discoveries, or new inventions.

Every discoverer who has tried to introduce his new idea and been rebuffed, every disciple of our school who has striven to practice the laws of the similars conscientiously has felt the truth of these observations keenly.

According to the view so presented we need not wonder that progress and improvement are so slow.

Let any gifted mind bring forth a grand conception of the mechanism of nature's works, and immediately, for the foregoing reasons, a storm of opposition is aroused. Later the storm is stilled, and a band of disciples gathers around, and then the idea has to endure yet another struggle for existence among its professed followers. A considerable number of these followers, incapable of a single original thought, but well equipped with that very common gift, censorious criticism, arrogantly dispute the terms of the discovery, impertinently offer to correct the great mind that conceived it, and boldly offer an emasculated substitute, thus making of it an object of scorn to all spectators.

Those of our school who industriously seek the simillimum, conscientiously apply it, and courageously wait its action in desperate cases, are so filled with the conviction of its truth and of its beneficent results that when they meet their conversation is an enthusiastic exchange of experiences. When they see their treasured principles hampered in progress, and themselves dishonored by the hostility of the dissenters in their own ranks, they may well exclaim in the language of Hamlet, but in the voice of sarcasm, "How noble in reason! in apprehension how like a god!"

